# Intention/Reflection (I/R) Practice Creates a Deeper APPE Connection for Student Pharmacists After COVID-19

**DOI:** 10.3390/pharmacy14020045

**Published:** 2026-03-05

**Authors:** Kerry K. Fierke, Gardner A. Lepp, Alina Cernasev

**Affiliations:** 1 College of Pharmacy, University of Minnesota, 1110 Kirby Drive, Duluth, MN 55812, USA; galepp@d.umn.edu; 2 College of Pharmacy, University of Tennessee Health Science Center, 301 S. Perimeter Park Dr., Suite 220, Nashville, TN 37211, USA; acernase@uthsc.edu

**Keywords:** I/R reflection, advanced pharmacy practice experience (APPE), qualitative, US, student pharmacists

## Abstract

(1) Background: In response to the educational challenges brought on by the COVID-19 pandemic, APPE preceptors implemented the Intention/Reflection (I/R) practice as a structured engagement tool. I/R is designed to promote engagement, motivation, metacognitive growth, and self-awareness among student pharmacists with the goal of enhancing learning experiences in diverse APPE settings. This project aimed to assess the impact of I/R strategies on student pharmacist engagement during APPEs in the post-pandemic landscape, with the overarching goal of identifying and advancing best practices in experiential pharmacy education. (2) Methods: This retrospective qualitative study included 20 student pharmacists from two U.S. colleges who participated in APPE elective rotations featuring I/R activities. Student pharmacists’ responses to five structured I/R prompts were collected and thematically analyzed by two independent researchers using qualitative data analysis software. (3) Results: Four themes were identified in the I/R responses: two themes each from the intention and reflection responses. The intention themes “Embracing Discomfort as a Catalyst for Confidence, Engagement, and Leadership Growth” and “Purposeful Precision: Growing into Adaptive Leadership” both illustrate the students’ journeys as they develop greater confidence and resilience in overcoming challenges. The reflection themes “Reflection as a Catalyst for Professional Learning and Engagement” and “Reflection as a Tool for Focused Growth and Self-Awareness” synthesized the evolution of the student pharmacist and forward thinking for future career. (4) Conclusion: Overall, participants perceived the I/R practice as transformative, citing benefits such as sustained learning, increased confidence, and continued professional development. These findings suggest that integrating I/R into experiential pharmacy education can significantly enhance student engagement and contribute to best practices for post-pandemic pharmacy training.

## 1. Introduction

The COVID-19 pandemic has profoundly altered nearly every facet of human life, with the education system experiencing particularly significant disruption [1]. While the long-term consequences for educational and academic outcomes may still impact students, it is clear that the pandemic has accelerated lasting changes in how learning is delivered and experienced [2]. Subsequently, educational institutions across the United States—including schools, colleges, and universities—implemented a range of adaptive measures. A systematic literature review investigated the effects of the COVID-19 pandemic on pharmacy education, specifically focusing on the transition to distance and online learning. Muhaimin et al., 2023 identified the benefits, challenges, and recommendations associated with this shift [3]. Key findings indicate that while the move to online education exposed systemic flaws, it also served as a catalyst for curriculum improvement, fostering communication, cost-effectiveness, and technological integration [3]. The review concludes with recommendations for future didactic decisions, emphasizing the need for adaptable online pedagogical approaches, sustained student motivation, and accessible online learning platforms [3]. The pandemic ushered in sweeping transformations in education, including the widespread adoption of remote learning platforms, smaller class sizes, reduced in-person contact, and strict limitations on campus visitors [4,5]. These innovative measures were implemented to protect health while ensuring the continuity of education [4,5]. Many of these strategies have endured, reshaping the landscape of daily didactic experiences. Today, blended online and in-person classes have become a defining feature of the new educational norm. In the same vein, a narrative review documents the adaptations made to experiential pharmacy education during the COVID-19 pandemic, specifically detailing the virtual training at the Lebanese International University and reviewing experiences from other global pharmacy colleges [6]. It highlights how virtual training successfully achieved desired learning outcomes, serving as a viable alternative to on-site rotations and demonstrating the importance of new services and expanded programs [6]. The study describes the use of platforms such as Google Classroom and Google Meet for activities such as virtual case discussions, patient monitoring, and order screenings, while acknowledging the challenge of absent eye contact [6]. Recommendations include diversifying online training platforms, incorporating more patient-centered activities, providing live virtual patient encounters, and adopting a blended learning approach for improved future training outcomes [6].

In the field of pharmacy education, student pharmacists faced unprecedented challenges as their didactic and experiential learning were abruptly disrupted by the pandemic. In the United States, pharmacy students typically complete three years of rigorous classroom instruction, followed by a final year focused on Advanced Pharmacy Practice Experiences (APPE). However, the COVID-19 pandemic forced many students to forfeit valuable clinical exposure during their APPEs and adapt to rapidly changing experiential rotation formats, fundamentally altering their educational journey.

In response to these disruptions, pharmacy educators and student pharmacists had to address new challenges related to isolation and disengagement. The Intention/Reflection (I/R) practice provides a framework to address these changes by encouraging engagement, motivation, and persistence, which positively affects metacognition and self-awareness [7,8]. I/R is a tool that can be used by preceptors in any type of APPE to provide a more personalized and engaging learning experience for pharmacy students [8].

### Intention/Reflection Practice

Reflection has long been recognized by educators as an essential tool for fostering deeper student engagement in the learning process. Through reflection, students can reevaluate their learning needs, develop self-awareness, strengthen critical thinking skills, build confidence, and shape their professional identities [9,10,11]. As Dewey famously asserted, “we do not learn from experience … we learn from reflecting on experience” [12]. Numerous models and approaches have been developed to promote reflective thinking. Yet, nearly all share what Dewey identified as the foundation of reflection: “active, persistent and careful consideration of any belief or supposed form of knowledge” (p. 118) [12]. Reflection challenges us to analyze our experiences in relation to our beliefs, ideas, and assumptions, with the goal of gaining new insights and informing our future decisions and perspectives.

However, reflective thinking activities are not a panacea and present several challenges, especially in the current era of artificial intelligence. Concerns about the authenticity of student submissions have persisted for years [13]. Additionally, unclear or confusing expectations from instructors may lead students to write what they believe the teacher wants to hear rather than offering genuine reflection (page 22) [14]. Despite these obstacles, supporting students in developing reflective thinking skills remains highly beneficial for learners.

Reflection is often thought to occur after a learning experience, yet growing evidence shows that students can also benefit from engaging in reflection before the experience begins. Knowles (1970) emphasizes that adult learners, unlike their younger counterparts, value the opportunity to draw on their prior experiences and knowledge, finding greater engagement when this existing foundation is applied to new contexts [15]. This approach encourages adult learners to ask insightful questions and to make meaningful connections between new information and authentic, real-world scenarios [15]. In this light, answering the question “Why are we learning this?” becomes essential for adult (i.e., college) learners [15]. Brookfield (2002) further supports this perspective, demonstrating that when adult learners are empowered to identify their own learning goals, their engagement and interest increase significantly [11]. The characteristics highlighted by Brookfield and Knowles such as self-direction, intrinsic motivation, and critical thinking indicate that advanced students achieve better outcomes when they can discern the purpose and relevance of their learning, and connect it directly to their professional development [11,15].

Much of the foundational research referenced above draws on Piaget’s model of learning (1961/1969) [16]. Piaget observed that knowledge acquisition is most effective when students are actively and intentionally engaged with the subject matter, as opposed to passively absorbing and memorizing isolated facts [16]. This insight laid the groundwork for constructivism, which posits that learners who interact meaningfully and authentically with new information are more likely to retain and apply it [16]. They do so by “constructing” knowledge in ways that integrate with their existing mental frameworks and experiences [16].

**Figure 1:**
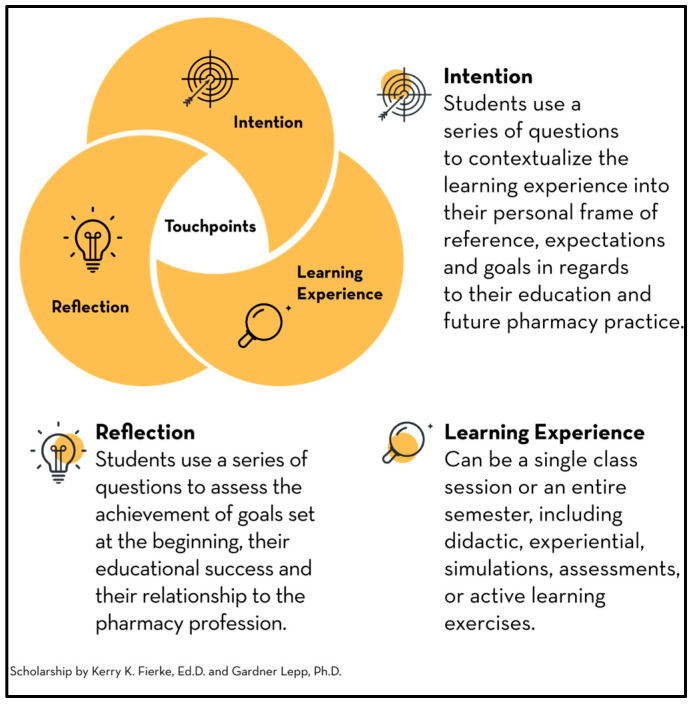
Description of Intention/Reflection practice.

Informed by the research above, the Intention/Reflection (I/R) practice was created to deepen student engagement (Figure 1). Rather than limiting reflection to the end of a learning experience, such as a class session, lab, lecture, course, or experiential activity, the I/R practice encourages students to identify their personal interests and articulate their learning objectives at the outset. This student-centered strategy leverages individual interests as a source of motivation and engagement. The process typically begins alongside the introduction of instructor-defined learning objectives, with students responding to three to five open-ended prompts, which invite them to consider why the learning experience matters to them, what they hope to achieve, and how they plan to reach their goals. At the conclusion of the experience, students revisit a similar set of prompts to assess the extent to which they met their objectives and to consider ways to enhance future learning experiences. This reflective process usually requires only 5–15 min at both the start and end of an activity, depending on its complexity, and can be easily implemented with minimal technology or simply pen and paper. Crucially, this practice does not replace instructor-determined learning objectives. Instead, it harnesses students’ intrinsic interests to foster a deeper psychological connection to the course content.

Building on the insights from previous I/R studies, Fierke et al. (2019), argue that the transition from APPE experiences to career options demonstrates how reflective learning translates into tangible professional pathways for pharmacy graduates [8]. Career options in the U.S. following APPE are remarkably diverse, enabling pharmacy graduates to pursue paths that reflect their unique interests, experiences, and skills [17]. APPE rotations provide students with hands-on exposure to various fields, including community pharmacy, hospital and clinical pharmacy, ambulatory care, industry, research, regulatory affairs, and managed care. Students who enjoy direct patient interaction often pursue roles as community or clinical/hospital pharmacists, while those drawn to research, drug development, or business may gravitate toward positions in the pharmaceutical industry or healthcare management. These APPE experiences not only help students identify their strengths but also allow them to build professional networks and develop practical skills, ensuring they are well prepared to enter the U.S. healthcare workforce or undertake further specialization through residencies or fellowships [18]. Wiley explores how APPEs foster professional growth, enhance the application of clinical knowledge, and prepare students to become “practice-ready,” emphasizing the significance of experiential learning in shaping successful pharmacy careers [18].

The I/R practice offers different advantages such as low time investment, low or no technology intensity, and no financial cost associated [7,8]. Thus, the aim of this project was to evaluate how I/R strategies influence student pharmacist engagement during APPEs in the after COVID-19, with the broader objective of identifying and promoting best practices for experiential education.

## 2. Materials and Methods

A retrospective qualitative study was conducted across two U.S. institutions. Student pharmacists from the University of Minnesota College of Pharmacy (UMN) participated in five-week APPE elective rotations, while students from the University of Tennessee Health Science Center (UTHSC) College of Pharmacy engaged in one-month elective rotations. Across both programs, a total of 20 students worked with two faculty members as part of the I/R practice. Distinctly, UMN students were enrolled in the Leadership Emphasis Area, a program characterized by a two-year partnership with faculty, completion of four specialized elective courses focused on leadership, and participation in an extensive leading change experience. This sustained engagement fostered strong relationships and a shared history of leadership development between UMN faculty and students. In contrast, UTHSC faculty met their students for the first time at the onset of the APPE rotation, with no prior relationship established before the APPE experience.

The I/R open-ended intention questions are administered on the first day of the APPE, immediately after students are introduced to the rotation’s activities and objectives. These responses are reviewed by the preceptor and revisited throughout the APPE, serving as ongoing reference points to reinforce student-preceptor connection and to align learning experiences with the desired outcomes. At the conclusion of the APPE, students complete and submit reflection questions, providing an opportunity to assess personal growth and achievement of objectives over the course of the rotation.

A retrospective qualitative thematic analysis was employed to examine the instructional and reflective (I/R) practices documented by student pharmacists during their APPE rotations. This methodological approach enabled a comprehensive, in-depth exploration of students’ thoughts and reflections while preserving the authenticity of their learning environment and avoiding any influence on their performance [19]. Utilizing a qualitative design also allowed for the capture of rich, nuanced insights into how student pharmacists engaged within the academic setting, providing a deeper understanding of their experiences and professional development [19].

Student pharmacists on APPEs between 2023 and 2025 (graduating in 2024, 2025, and 2026) participated in the study. All data were de-identified prior to analysis to ensure confidentiality. Two questions were analyzed for intention: *(1) Knowing your personal areas of strength and preferences for learning, what obstacles do you expect to encounter during this rotation? How will you overcome those obstacles? (2) Provide your strengths and ways you can feel you can incorporate them into the rotation.* For the reflection, three questions were analyzed: *(1) Provide the ways that you have incorporated your strengths throughout the rotation. (2) How has this Intention/Reflection activity been helpful in enhancing your experience?* The third question analyzed was slightly different for each university due to the types of students and APPE type. One APPE was focused on leadership in academia and the other was research in academia therefore, these were slightly altered. *(3) What did you learn about yourself as a leader [student pharmacist]? How will you continue to develop your leadership skills as a professional? [skills as a professional pharmacist].*

A thematic analysis guided the interpretation and categorization of the answers of the student pharmacists documented during the academic APPE rotation. Following Braun and Clarke’s six-step framework, the research team first familiarized themselves with the data by repeatedly reading the reflections [20]. Using an inductive approach, one researcher (AC) coded all the data with Dedoose^®^ (Version 9.10.27, Los Angeles, CA, USA), a qualitative analysis software. The codes were developed to capture meaningful units of information that reflected the nature, context, and impact of student inquiry and reflection activities [20].

These initial codes were then merged and grouped into broader categories that represented patterns in how students engaged in tailoring their career goals, and enhancement of their skills. Through comparison and discussion among the research team members (AC, KF, GL), these categories were analyzed, leading to the emergence of themes that described the educational significance of inquiry/reflection activities and their contributions to professional identity formation [20].

To enhance the trustworthiness and rigor of the thematic analysis, the research team (AC, KF, GL) reviewed the codes and categories, resolving any discrepancies by reaching a consensus [21]. An audit trail was maintained using Dedoose^®^ to document analytical decisions. The resulting themes provided a comprehensive understanding of the depth and diversity of student pharmacists’ reflections and recommendations within the academic APPE environment [21].

An Institutional Review Board (IRB) approved this study as exempt (University of Tennessee Health Science Center (IRB #24-09820 XM, 24 September 2024) and University of Minnesota (IRB #00026976, 18 December 2025)).

## 3. Results

A total of twenty student pharmacists I/R were coded and resulted in the identification of four themes: two themes each from the intention and reflection responses. The intention themes: “Embracing Discomfort as a Catalyst for Confidence, Engagement, and Leadership Growth” and “Purposeful Precision: Growing into Adaptive Leadership” both illustrate the students’ journeys as they develop greater confidence and resilience in overcoming challenges. The reflection themes: “Reflection as a Catalyst for Professional Learning and Engagement” and “Reflection as a Tool for Focused Growth and Self-Awareness” synthesized the evolution of the student pharmacist and forward thinking for future career.

### 3.1. Intention Themes

The first intention theme “Embracing Discomfort as a Catalyst for Confidence, Engagement, and Leadership Growth” describes how student pharmacists recognized that stepping outside their comfort zones was essential for personal and professional development. This theme reflects the students’ belief that embracing discomfort and unfamiliar situations can serve as a catalyst for building confidence, deepening engagement, and fostering leadership development. Student pharmacists recognized personal obstacles, including hesitation, self-doubt, and challenges in moving from planning to action, especially in new or high-pressure environments.

The following quotes illustrate how student pharmacists identified how they will overcome these obstacles, student pharmacists deliberately employed strategies such as active listening, seeking clarification, maintaining adaptability, and fully participating even when feeling uncertain.

One student shared how they were planning to: “… work more on active listening so I can be truly engaged in the conversations we have, and I am going to overcome my challenges by taking time to ask clarifying questions.” Whereas another student noted how to overcome obstacles: “enter each day with an open mind, making sure to stay flexible, listening carefully, and embracing the unknown as part of this learning process. By doing this, I can grow as a leader and person and become better at problem solving and connecting with the people around me daily.”

Perhaps more insightfully, some students shared the importance of mindfulness throughout the uncertain experiences. As one student stated: *“As I am put into new situations and work on projects, I am unfamiliar with, I want to be very mindful about fully participating even if I don’t know all the answers.”* Additional examples of student comments are included in Table 1.

Several student intentions highlighted the use of leaning into discomfort as a learning strategy. Student pharmacists described proactively placing themselves in unfamiliar environments to challenge their limits, acquire new skills, and broaden their knowledge, viewing such experiences as essential for professional growth. For example, one student pharmacist states the following: *“One of my personal areas of strengths and learning preferences is putting myself in uncomfortable/unfamiliar situations with the goal to challenge myself and/ or learn a new skill.”*

Strong interpersonal and communication skills consistently emerged as key tools for students, facilitating engagement, collaboration, and meaningful connections amid uncertainty. Furthermore, preparation and self-reflection were identified as vital tools for building confidence, particularly in clinical and discussion-based settings, empowering individuals to express themselves more effectively and minimize self-doubt. This empowerment is reflected in the student quote: *“I am quick to second guess myself when answering clinical questions … To overcome the barrier of clinical confidence, I will adequately prepare for topic discussions in advance so that if questions arise, I will answer them for myself prior to topic, so I am confidently able to articulate a response by the time of topic discussion.”*

The second theme “Purposeful Precision: Growing into Adaptive Leadership,” highlights how students learn to lead with intention and flexibility. Through the intention practice students noted a targeted skill-building approach on their APPEs to demonstrate growth in strategic thinking and adaptive leadership. The theme of purposeful precision emphasizes an intentional effort to grow in confidence, adaptability, and leadership. This theme highlights how precision is both a strength and a challenge, shaping how tasks are completed, decisions are made, and leadership skills are developed.

Underlying this theme includes being detail-oriented and focused. This is evident in the ability to apply precision to academic and professional tasks, such as being *“detail-oriented so being able to use that when analyzing manuscripts.”* Similarly, strong focus, attention to detail, and determination allow tasks to be completed “efficiently while also correctly,” with motivation to *“finish strong and end on a good rotation”.* Time management further reinforces this precision, including having “*good time management skills*” and creating “*a project tracker to ensure everything is completed and done on time.*”

However, purposeful precision also includes awareness of its limitations. Being reflective and detail-oriented can sometimes lead to “*overanalyzing decisions or hesitating to act quickly,*” which may hinder confident leadership in fast-paced environments. Additionally, stepping into leadership roles alongside more experienced professionals can trigger imposter syndrome, presenting another internal obstacle to confidence. There is also recognition that external responsibilities including board preparation and graduation that may challenge time management during the rotation. One student pharmacist says: “*Another obstacle will be the outside responsibilities that are co-occurring during this rotation. In this season of board preparation and graduation, I could see time management being difficult since there are so many tasks to complete outside of rotation in order to successfully graduate.*”

To address these challenges, some students develop purposeful strategies. Focusing solely on rotation duties during rotation hours, silencing distractions, and working far ahead of deadlines are planned approaches to maintain efficiency and balance competing responsibilities. These strategies allow precision to remain productive rather than overwhelming. As one student pharmacist further elaborates: “*To overcome potential time management issues, I will solely focus on rotation duties during the rotation hours, that way I am able to confidently complete all the tasks of the rotation in a timely manner. I will also limit distractions during rotation time by silencing my cell phone during rotation hours. Additionally, I will make sure to work far ahead of due dates for tasks such as journal clubs and the presentation so that I have time for my other commitments throughout the month.*”

Learning style and growth further support this theme. Learning best through observation and examples before attempting tasks independently reflects a thoughtful and methodical approach. Visual organization tools such as “lists, tables, or concept maps” help track information and enhance understanding. One student pharmacist asserts that: *“Additionally, I typically learn best by observing or being shown examples of what is expected of me before attempting myself. I do sometimes like to attempt projects without guidance since I know in the future there may be situations where guidance is not given.”*

At the same time, there is a desire to grow by attempting projects without guidance and by contributing ideas more freely, even when there is uncertainty. Holding back ideas unless they are right limits students from speaking up. As one student shared: *“I find that I usually will not give suggestions or ideas unless I know they are correct or “good”. This can cause me to not be as engaged in the creative process as I want to be. I want to overcome this barrier by saying my ideas out loud more often.”* Showing they are ready to contribute without hesitation.

Finally, strong problem-solving skills anchor this theme. Using intuition to “attack any situation” and identifying as a “well-rounded student with a wide bandwidth” demonstrates adaptability and confidence beyond traditional academic measures. One student pharmacist explains: *“As we discussed throughout the week, I am not always the most “book smart” person, but I can usually use my intuition to attack any situation. I consider myself to be a well-rounded student with a wide bandwidth and ability to accomplish a variety of tasks.”*

### 3.2. Reflection Themes

The reflection theme “Reflection as a Catalyst for Professional Learning and Engagement” highlights reflection as a vital driver for impactful professional growth and development. By purposefully setting clear intentions, participants discovered a deeper sense of direction in their career and learning journeys, engaging in activities with heightened purpose, focus, and accountability. Reflective practices seemed to empower student pharmacists to celebrate personal growth, embrace transformative shifts in mindset, and appreciate the diverse range of skills gained throughout the rotation. One student pharmacist stated: *“Thinking about my intentions has helped me create a firmer foundation for what I am setting out to do, and reflecting on each experience has also helped me to be more aware of what I have achieved and also get in touch with how each experience has made me feel in order to learn and grow along the way.”* In addition, this highlights how I/R revealed learning and growth that might otherwise go unrecognized. Similarly, another student emphasized that *“intentions set the tone for the day”* and increased the likelihood of achieving desired learning outcomes by establishing clear goals in advance. Table 2 further explores the student comments on the themes.

Accountability and sustained engagement emerged as additional outcomes of intentional reflection. One student pharmacist explained, *“By setting an intention I knew I would have to reflect on the experiences I embarked on, so I made sure to be engaged,”* illustrating how reflection motivated active participation and follow-through. Other student pharmacists described the intention component as a reference point for monitoring progress and revising goals. In particular, one student noted: *“In addition, the intention portion of this activity served as a resource for me to look back on throughout the rotation to ensure I was making forward progress and a way to restructure or reformat my goals to support my intentions.”* The student responses also emphasize how I/R enhanced self-awareness and documented professional development. Participants reported that reflecting helped them recognize mindset shifts, skill acquisition, and the overall value of the rotation. One student stated: *“Having a record of my accomplishments makes me realize how incredibly beneficial this rotation has been*.” Collectively, these reflections illustrate how intentional reflection functions as a catalyst for purposeful learning, enhanced engagement, and sustained professional development across experiential learning environments.

Finally, several student pharmacists described transferring reflective practices beyond the rotation itself. One individual shared, *“I actually started using this activity to debrief after each Residency Interview,”* demonstrating how intentional reflection supported continuous learning and professional preparation. Another student pharmacist highlighted that I/R encouraged *“deeply and intentionally reflection my own skill development with tangible examples”* that could be referenced in future professional contexts. Others emphasized that reflecting on intentions helped them become more aware of achievements, emotional responses, and areas for improvement, reinforcing reflection as an ongoing tool for growth and development.

The second theme “Reflection as a Tool for Focused Growth and Self-Awareness” reflects a transformative framework which prompts learners to take charge of their growth, remain accountable, and celebrate both personal and professional achievements. By setting clear intentions at the beginning of the APPE, participants seemed to channel their energy and remain engaged with their unique goals. The practice of reflection appeared to provide meaningful opportunities to acknowledge progress, embrace mindset shifts, and identify new avenues for development. Together, I/R appeared to foster deeper self-awareness, strengthen learning, and empower participants to approach their rotation with confidence, gratitude, and a strong sense of purpose.

As students shared in the I/R practice, they explain the way it helped them hone direction on important or valuable experiences in the APPE. For example, one student pharmacists noted that I/R: *“helped me understand where I wanted to focus my energy.”* It was also used by another student *“to ground myself and focus on what I really want to accomplish.”* As another student shared these are critical to get the most out of the experiences: *“Intentions set the tone for the day. I was more likely to get what I wanted out of the activities I did by thinking about what I wanted to learn beforehand.”*

Mindset was another recognition for self-awareness and growth as students shared the ways in which I/R facilitated an opportunity for them to recognize their own changes. This was noted by several students throughout their own personal examination of their I/R documents. Additionally, it identified confidence and gratitude as described by a student, *“Reading my Intention from the beginning of the month and now reflecting on the end of the month has me feeling grateful and confident in the way that I used my time … it’s always helpful to stop and reflect…to acknowledge areas of growth… but also to give myself a moment to be proud of how far I have come.”* Positive sentiments such as these were incorporated throughout the statements provided by the students.

## 4. Discussion

Overall, the student pharmacists expressed positive gains by incorporating the I/R experience on their APPEs, as demonstrated in themes identified in the student comments. In particular, students valued the practice of documenting and referencing their own personal learning goals.

Our findings demonstrate that “Reflection as a Catalyst for Professional Learning and Engagement” and “Reflection as a Tool for Focused Growth and Self-Awareness” plays a pivotal role in the evolution of student pharmacists and their forward-thinking approach to future careers. This aligns closely with the study by Quinn et al. (2020), conducted in the United Kingdom, which employed a grounded theory methodology to investigate the lived experiences of MPharm students during early preregistration training placements, with a particular focus on professional identity development [22]. Quinn et al. identified “developing a professional identity” as a central process, encompassing stages such as reflection, selection of desirable attributes, professional socialization, and evolving perceptions of the pharmacist’s role [22]. Their findings highlight the importance of early work-based placements in nurturing students’ sense of professional identity, increasing motivation for learning, and shaping aspirations for their future professional selves [22]. In light of these findings, our results contribute to the growing body of evidence supporting the integration of structured reflective practice in pharmacy education [22]. The parallel between our I/R and those of Quinn et al. highlight the universal value of reflection in fostering professional growth, engagement, and identity formation among student pharmacists globally [22].

The integration of I/R into pharmacy education not only aligns with ACPE Standards particularly in fostering individualized mentoring and targeted professional development, but also serves as a critical catalyst for enhancing student engagement and identity formation [23]. According to Standard 3.3.e, I/R enables preceptors to tailor their mentoring approaches based on students’ individual aspirations and learning objectives for APPEs [23]. Although incorporating I/R does not directly alter the prescribed learning objectives of APPEs, it meaningfully enriches the learning experience by fostering deeper student participation and motivation.

The findings of an Australian ethnographic study further contextualize the significance of such approaches [24]. The study, conducted over four weeks in an undergraduate pharmacy program, revealed that a curriculum centered primarily on technical knowledge may inadvertently limit students’ opportunities to develop a professional self-concept [24]. Students reported few chances to engage with professional role models, experiment with the pharmacist role, or critically reflect on their evolving professional identities [24]. Instead, the traditional curriculum risked reinforcing students’ perceptions of themselves as learners rather than as future practitioners, leaving their understanding of professional practice underdeveloped [24]. Taken together, these insights highlight a gap between the acquisition of technical skills and the formation of professional identity [24]. The literature suggests that pharmacy educators should reconsider curricular design, emphasizing patient-centeredness, practice integration, authentic role modeling, and practical experimentation. By incorporating these elements, programs can better support the holistic development of students’ professional identities and prepare them for meaningful practice. This discussion demonstrates the need for curricular reform that bridges the divide between knowledge and professional self-concept, with I/R serving as a promising mechanism to achieve these goals.

The theme of “Growing into Adaptive Leadership” is, Heifetz argues, vital for student pharmacists [25]. The work of Heifetz et al. focuses on “adaptive challenges,” which require learning, experimentation, and changes in values/habits, rather than technical problems that can be solved by expertise alone [25]. Overall, this theme highlights that true growth flourishes not by avoiding uncertainty, but by boldly seeking it out. When learners lean into discomfort, fuel their curiosity, and dive wholeheartedly into the unknown. For example, even without all the answers, the student pharmacists unlock transformative opportunities. Challenges become springboards for surging confidence, inventive problem-solving, and the emergence of authentic, resilient leaders.

When we look across the four primary themes identified in the data, we see several hidden but extremely important educational concepts and theories at work. One of those concepts is engagement. While engagement can be difficult to track definitively by grades, or even classroom participation [26], the student data from the I/R practice seems to indicate that students experience increased levels of engagement when I/R is employed. Engagement in education and the development of self-awareness were important in shaping student pharmacists’ attitudes during APPE elective rotations. Through active participation and reflection, students moved beyond viewing their rotations as mere requirements to be completed. Instead, they began to see these experiences as opportunities for personal and professional growth, fostering a sense of connection and investment in their learning. This shift was not only about fulfilling tasks but also about becoming more engaged with the learning process, collaborating with peers and mentors, and seeking feedback to improve. By developing greater self-awareness, students were able to identify their strengths and areas for improvement, set meaningful goals, and take ownership of their learning journey [27]. As a result, the APPE rotations transformed from a transactional process into an integrated, enriching experience that contributed to students’ overall development.

These findings can be viewed through both their limitations and strengths. One limitation inherent in this reflective writing activity is that student pharmacists completed the I/R as part of an APPE activity. This context may have influenced their responses, as they were aware that their preceptor would review their work.

This awareness could shape not only what students choose to share but also the depth and authenticity of their reflections. As the data are based solely on the I/R outcomes, the findings may not fully capture student pharmacists’ genuine perspectives. Nevertheless, while APPEs were elective and may have attracted students already interested in these experiences, the integration of structured, longitudinal I/R activities within authentic APPE settings offers valuable insight into student pharmacists’ developing professional identities in real-world practice. The recurring themes identified across APPE student pharmacists at two different U.S. institutions demonstrate the strength of this rich data. Despite potential predispositions, the I/R process appeared to foster meaningful engagement and deep reflection, indicating its value as an educational strategy that transcends initial student interest.

One important strength is that the activities were implemented at two distinct U.S. Colleges of Pharmacy, enhancing both the robustness and generalizability of the findings across institutional contexts. Notably, while UMN student pharmacists were concurrently enrolled in a formal leadership course, their counterparts at the UTHSC were not. This allowed the I/R practice to be evaluated across varying curricular structures and differing levels of prior exposure to reflective or leadership-focused learning.

## 5. Conclusions

I/R has been shown across multiple research initiatives to benefit student learning outcomes. Preceptors can continually encourage engagement by incorporating an I/R practice into APPEs. The findings from this study highlight that student pharmacists identify I/R as a meaningful practice that transforms experiences into sustained learning, confidence, and professional development.

## Figures and Tables

**Table 1:** Intention themes with student comments.

**Theme 1: Embracing Discomfort as a Catalyst for Confidence, Engagement, and Leadership Growth**	
Student Comment	I am going to work more on active listening so I can be truly engaged in the conversations we have, and I am going to overcome my challenges by taking time to ask clarifying questions. Rather than just nod my head and agree with things, I will make sure I slow down and ask myself if I understand what was just said. To overcome my obstacles of not being able to visualize ideas in my mind, I am going to ask for some time to think about things before answering and ask clarifying questions.
Student Comment	To overcome this obstacle, I have to enter each day with an open mind, making sure to stay flexible, listening carefully, and embracing the unknown as part of this learning process. By doing this, I can grow as a leader and person and become better at problem solving and connecting with the people around me daily.
Student Comment	One of my personal areas of strengths and learning preferences is putting myself in uncomfortable/unfamiliar situations with the goal to challenge myself and/ or learn a new skill. I believe my personable personality and people skills are also strengths that assist with my prior mentioned strength/learning preference.
Student Comment	My biggest strengths are my people skills and my willingness to put myself into unknown/uncomfortable situations in order to become comfortable/knowledgeable. I can incorporate these strengths into this rotation by asking to be part of projects I’m unfamiliar with or asking for additional resources to educate myself.
Student Comment	This makes it hard to transition from the planning stage to the action stage of many things, even something as simple as giving feedback. As I am put into new situations and work on projects I am unfamiliar with I want to be very mindful about fully participating even if I don’t know all the answers.
Student Comment	One of the obstacles I expect to encounter during this rotation is clinical confidence. I am quick to second guess myself when answering clinical questions in topic discussions. I have made significant improvements in building clinical confidence through previous APPEs; however, this is always an area of improvement for me. UT #4: I am dedicated to overcoming these obstacles. To overcome the barrier of clinical confidence, I will adequately prepare for topic discussions in advance so that if questions arise, I will answer them for myself prior to topic so I am confidently able to articulate a response by the time of topic discussion.
Student Comment	One of my personal areas of strengths and learning preferences is putting myself in uncomfortable/unfamiliar situations with the goal to challenge myself and/ or learn a new skill. I believe my personable personality and people skills are also strengths that assist with my prior mentioned strength/learning preference.
Student Comment	I expect to struggle with confidence and action. One of my top 5 strengths is learner. For me, this means that I like to have all the information and ample opportunity to review before presenting or sharing something with others.
**Theme 2: Purposeful Precision—Growing into Adaptive Leadership**	
Student Comment	My personal strengths include being detail-oriented and reflective, which may sometimes lead to overanalyzing decisions or hesitating to act quickly. In a fast-paced environment, this could hinder my ability to lead confidently. Another potential obstacle is managing imposter syndrome when stepping into a leadership role alongside more experienced professionals.
Student Comment Student Comment	I am detail-oriented so being able to use that when analyzing manuscripts. I have good time management skills, so creating a project tracker to ensure everything is completed and done on time. My strengths are focus, attention to detail, and determination. I think this will help me on this rotation because it will allow me to get tasks done efficiently while also correctly. In addition, I am always determined to do my best on rotations. As this is my last rotation, I am excited to finish strong and end on a good rotation. I am looking forward to a great month!
Student Comment	Additionally, I typically learn best by observing or being shown examples of what is expected of me before attempting myself. I do sometimes like to attempt projects without guidance since I know in the future there may be situations where guidance is not given.
Student Comment	Another strength that I have is my problem-solving ability. As we discussed throughout the week, I am not always the most “book smart” person, but I can usually use my intuition to attack any situation. I consider myself to be a well-rounded student with a wide bandwidth and ability to accomplish a variety of tasks.
Student Comment	I learn well when I can create a visual of some sort (list, table, concept map) that helps me keep track of information.
Student Comment	I find that I usually will not give suggestions or ideas unless I know they are correct or “good”. This can cause me to not be as engaged in the creative process as I want to be. I want to overcome this barrier by saying my ideas out loud more often.
Student Comment	Another obstacle will be the outside responsibilities that are co-occurring during this rotation. In this season of board preparation and graduation, I could see time management being difficult since there are so many tasks to complete outside of rotation in order to successfully graduate.
Student Comment	To overcome potential time management issues, I will solely focus on rotation duties during the rotation hours, that way I am able to confidently complete all the tasks of the rotation in a timely manner. I will also limit distractions during rotation time by silencing my cell phone during rotation hours. Additionally, I will make sure to work far ahead of due dates for tasks such as journal clubs and the presentation so that I have time for my other commitments throughout the month.

**Table 2:** Reflection themes with student comments.

**Theme 1: Reflection as a Catalyst for Professional Learning and Engagement**	
Student Comment	Intention/reflection allowed me to understand the why behind the activities we do.
Student Comment	Reflecting led me to recognize mindset changes as I completed the rotation. Intentions set the tone for the day. I was more likely to get what I wanted out of the activities I did by thinking about what I wanted to learn beforehand. Intention/reflection also kept me accountable.
Student Comment	By setting an intention I knew I would have to reflect on the experiences I embarked on, so I made sure to be engaged and actually complete the intentions I set so I was able to complete a reflection.
Student Comment	In addition, the intention portion of this activity served as a resource for me to look back on throughout the rotation to ensure I was making forward progress and a way to restructure or reformat my goals to support my intentions.
Student Comment	This intention/reflection activity has helped enhance this learning experience as it encouraged me to deeply and intentionally reflect on my own skill development with tangible examples that I can reference when necessary. This activity has also allowed me to tailor my learning experience and focus on certain areas of leadership based on my personal interests and objectives for this rotation.
Student Comment	Reflecting has also reminded me of the vast topics and skills that I’ve attained knowledge about throughout this time. Having a record of my accomplishments makes me realize how incredibly beneficial this rotation has been.
Student Comment	The I/R activity helps to enhance my rotation experience by helping me outline what I’d like to achieve during the rotation and how I could accomplish this. It also helps me reflect on how I work best, what I achieved, and what I could improve for next time.
**Theme 2: Reflection as a Tool for Focused Growth and Self-Awareness**	
Student Comment	It provided me with a focus for the rotation and helped me understand where I wanted to focus my energy. Setting intentions and taking time to look back at them from time to time helped me understand what I still needed to work on for the remainder of the rotation. Reflecting on the rotation helped me realize how much I have grown, but also where I need to develop.
Student Comment	It also allowed me to recognize my growth, at times I didn’t realize how much I had learned.
Student Comment	By setting an intention I knew I would have to reflect on the experiences I embarked on, so I made sure to be engaged and actually complete the intentions I set so I was able to complete a reflection.
Student Comment	The intention was helpful to ground myself and focus on what I really want to accomplish. I am not sure if I would have been as intentional throughout the rotation if I had not completed the intention. The reflection was helpful to remind myself all that I have completed and note if there were any changes that occurred to my intentions during the rotation.
Student Comment	This intention/reflection activity has helped enhance this learning experience as it encouraged me to deeply and intentionally reflect on my own skill development with tangible examples that I can reference when necessary. This activity has also allowed me to tailor my learning experience and focus on certain areas of leadership based on my personal interests and objectives for this rotation.
Student Comment	This activity has been helpful in focusing my attention and efforts to tailoring this rotation to my own goals. It has also allowed me to recall all of the various experiences I participated in, and reflect on my key takeaways. I plan to incorporate Intention/Reflections in my rotations going forward to direct and track my growth!
Student Comment	Intention/Reflection has helped me center my thoughts and truly focus on what I wanted to develop on this journey, and also think back to all the things I was able to accomplish and how to improve and carry it with me moving forward.
Student Comment	Thinking about my intentions has helped me create a firmer foundation for what I am setting out to do, and reflecting on each experience has also helped me to be more aware of what I have achieved and also get in touch with how each experience has made me feel in order to learn and grow along the way.
Student Comment	I feel like I reached all of my goals for the month and was able to get everything I wanted to experience out of the rotation. It was truly a pleasure to be able to take this month to grow into a more confident researcher and medical writer. I know that the skills I learned this month will help me be successful in my career. Had I not done this Intention/Reflection activity, I would not have reflected this deeply into the experience and may have not realized all of the ways I learned and grew this month.
Student Comment	This Intention/Reflection activity has been helpful in allowing me to reflect on the areas in which I was to continue to hon as I continue on in my career. It’s always helpful to stop and reflect/ be introspective not only to acknowledge area of growth.
Student Comment	I/R has been helpful in enhancing my experience during the rotation. I enjoyed each week completing a reflection because it allows you to examine yourself and see where you were and the progress you have made … It is also good to be self-aware in the future so you know where your strengths are and how you can be an asset as well as areas where you can improve or use the assistance of another team member.
Student Comment	I think that this Intention/Reflection activity has been very helpful in enhancing my experience … Reading my Intention from the beginning of the month and now reflecting on the end of the month has me feeling grateful and confident in the way that I used my time this past month.

## Data Availability

The data presented in this study are available on request from the corresponding author due to privacy or ethical restrictions.

## Abbreviations

The following abbreviations are used in this manuscript.

APPE	Advanced Pharmacy Practice Experience
I/R	Intention/Reflection
UMN	University of Minnesota College of Pharmacy
UTHSC	University of Tennessee Health Science Center College of Pharmacy
